# Glucagon in metabolic disease: a mini-review of emerging multi-organ roles beyond glycemic control

**DOI:** 10.3389/fendo.2025.1645041

**Published:** 2025-07-31

**Authors:** Seung Hee Lee, Hyeon Young Park, Ji Ho Yun, Eun Kyoung Do

**Affiliations:** Division of Endocrine and Kidney Disease Research, Department for Chronic Disease Convergence Research, Korea National Institute of Health (KNIH), Cheongju, Republic of Korea

**Keywords:** glucagon, glucagon resistance, α-cell dysfunction, hyperglucagonemia, metabolic diseases

## Abstract

Glucagon, once seen as an insulin counter-regulatory hormone, is now recognized as a key regulator of systemic energy balance, with expanding relevance across a spectrum of cardiometabolic diseases. While its traditional roles in liver glucose regulation are well established, new evidence highlights glucagon’s involvement in amino acid metabolism, fat oxidation, appetite control, heat production, and cardiovascular health. Nonetheless, the broader effects of glucagon imbalance, especially in cases of α-cell overactivity and glucagon resistance, are still not fully understood in chronic conditions like type 2 diabetes mellitus (T2DM), non-alcoholic fatty liver disease (NAFLD), chronic kidney disease (CKD), obesity, and hypertension. This mini-review consolidates current knowledge of glucagon signaling, highlighting its regulatory mechanisms, multi-organ metabolic functions, and emerging therapeutic approaches. We suggest that long-term changes in glucagon secretion could be an upstream factor driving diabetic complications affecting the liver, kidney, and cardiovascular system. By incorporating recent discoveries, we aim to establish a conceptual basis for future translational research on glucagon’s systemic effects within the framework of diabetic cardiometabolic dysfunction.

## 1 Introduction

Diabetes mellitus (DM) is a chronic metabolic disorder characterized by persistent hyperglycemia resulting from impaired insulin secretion, reduced insulin sensitivity, or functional deficiency. Among Korean adults aged ≥30 years, the prevalence of diabetes was 15.5% in 2021–2022 (1). In the United States, more than 28.5 million adults have been diagnosed with DM, while an estimated 8.5 million remain undiagnosed, and 96 million are classified as having prediabetes (2). Diabetes mellitus (DM) and related metabolic disorders remain among the leading global health burdens, with rising prevalence and substantial morbidity linked to hyperglycemia, dyslipidemia, and cardiovascular complications.

Historically, pathophysiologic models of diabetes have mainly focused on insulin deficiency or resistance, emphasizing β-cell dysfunction. However, recent research has shifted attention to the paracrine functions of other endocrine cells, especially cells and their preproglucagon-derived peptides (3). Glucagon, secreted from pancreatic α-cells, plays a crucial role in maintaining euglycemia by stimulating hepatic glucose output through the processes of gluconeogenesis and glycogenolysis. Beyond glucose regulation, glucagon influences amino acid metabolism, lipid oxidation, bile acid turnover, and thermogenesis. Disruptions in these regulatory pathways contribute to the pathogenesis of T1DM and T2DM (4–7) NAFLD, and CKD, often manifesting as hyperglucagonemia and hepatic glucagon resistance. These findings have led to a conceptual shift toward the “glucagonocentric hypothesis,” which posits that glucagon is not merely insulin’s counter-regulatory hormone, but also a driver of disease.

## 2 Discovery and physiological roles of glucagon

A century after its discovery, glucagon remains recognized as a key regulator of metabolic homeostasis. In 1922, Charles Kimball and John Murlin identified a pancreatic hormone that elevates blood glucose levels, naming it “glucose agonist” or simply “glucagon.” This landmark discovery initiated a century of intensive research and shaped our current understanding of glucagon physiology. While its hormonal properties were debated for several decades, it was ultimately purified and crystallized in 1953, and its 29-amino-acid sequence was identified in 1957. The development of a radioimmunoassay (RIA) by Roger Unger in 1959 enabled the precise measurement of glucagon concentration (8).

Glucagon primarily stimulates hepatic glycogenolysis and gluconeogenesis, resulting in elevated blood glucose levels (8). Its secretion decreases with carbohydrate-rich diets and increases with protein-rich meals, which also regulate amino acid metabolism (8, 9). Glucagon enhances hepatic uptake and breakdown of amino acids and increases ureagenesis, converting ammonia into urea (9–11). Disruption of the liver–α–cell axis can lead to hyperaminoacidemia, which then triggers excessive glucagon secretion (8, 12) In lipid metabolism, glucagon facilitates hepatic lipolysis, promotes cholesterol clearance, and triggers ketogenesis by stimulating the use of fatty acids (8, 13, 14). Additionally, glucagon affects the brain by decreasing food intake and helping regulate blood glucose through the central nervous system (8, 15). Glucagon receptors in the hypothalamus coordinate hepatic glucose production via neural pathways that help sustain overall metabolic balance (8, 16). Furthermore, glucagon-induced amino acid catabolism may contribute to muscle wasting, supplying substrates for gluconeogenesis and thereby perpetuating hyperglycemia (17).

## 3 Regulation of glucagon secretion

Glucagon secretion is tightly regulated by various stimuli, including nutrients such as glucose, amino acids, and fatty acids, as well as endocrine factors like somatostatin and neural inputs (8, 18). During hyperglycemic conditions, somatostatin released from pancreatic δ-cells inhibits glucagon secretion (8, 19, 20). In contrast, hypoglycemia promotes glucagon secretion through activation of the sympathetic nervous system and elevated circulating amino acid levels (8, 19, 20). Incretin hormones, including glucagon-like peptide-1 (GLP-1) and glucose-dependent insulinotropic polypeptide (GIP), affect glucagon release differently: GLP-1 suppresses it, while GIP promotes secretion (21). Stress hormones, such as epinephrine (also known as adrenaline) and cortisol, stimulate glucagon secretion as a compensatory mechanism to maintain glucose homeostasis during physiological stress (8, 21). The electrical activity of α-cells, influenced by ATP-sensitive potassium (KATP) channels and calcium influx, plays a key role in glucagon release (22, 23).

Besides these extracellular signals, α-cell secretory activity is carefully regulated by intracellular second messenger systems, primarily through cyclic adenosine monophosphate (cAMP) signaling (24). In pancreatic β-cells, cAMP enhances calcium signaling to promote insulin release, while in α-cells, cAMP acts as the primary trigger for glucagon secretion, with calcium serving as a secondary messenger. During stress or exercise, stimulation of the sympathetic nerve increases cAMP levels, triggering glucagon release by activating protein kinase A (PKA) and exchange protein directly activated by cAMP (Epac), which help activate calcium channels and vesicle trafficking. Additionally, cGMP-dependent PKA signaling phosphorylates essential calcium-regulating proteins, such as phospholamban (PLB) and troponin, which assist in calcium mobilization. Under hypoglycemic conditions, this cAMP–PKA–calcium pathway is essential for maintaining normal blood sugar levels.

Beyond cAMP, G protein–coupled receptor (GPCR) pathways also play a role in glucagon regulation. Activation of Gs-linked β-adrenergic receptors by catecholamines further increases cAMP levels, while Gq-coupled receptors, such as the vasopressin 1b receptor (V1bR), activate phospholipase C (PLC) and produce inositol trisphosphate (IP3), leading to increased intracellular calcium and enhanced glucagon release (25).

Glucagon secretion from pancreatic α-cells is also heavily regulated by intra-islet paracrine signals. The most essential inhibitory signals include insulin, somatostatin, γ-aminobutyric acid (GABA), and zinc, all of which are secreted by neighboring β- and δ-cells. Insulin interacts with its receptor on α-cells to inhibit exocytosis through PI3K–Akt–dependent pathways (24, 25). Somatostatin binds to somatostatin receptor subtype 2 (SSTR2), reducing cAMP and calcium levels, which suppresses glucagon secretion (19, 20). GABA activates GABAA receptors on α-cells, leading to chloride influx and membrane hyperpolarization, which subsequently inhibits α-cell activity. Zinc, co-secreted with insulin, influences KATP channels to promote hyperpolarization, thereby further stabilizing the membrane potential (7). Disruption of these inhibitory pathways—common in type 2 diabetes mellitus (T2DM)—leads to paradoxical hyperglucagonemia and impaired glycemic control.

**Figure 1:**
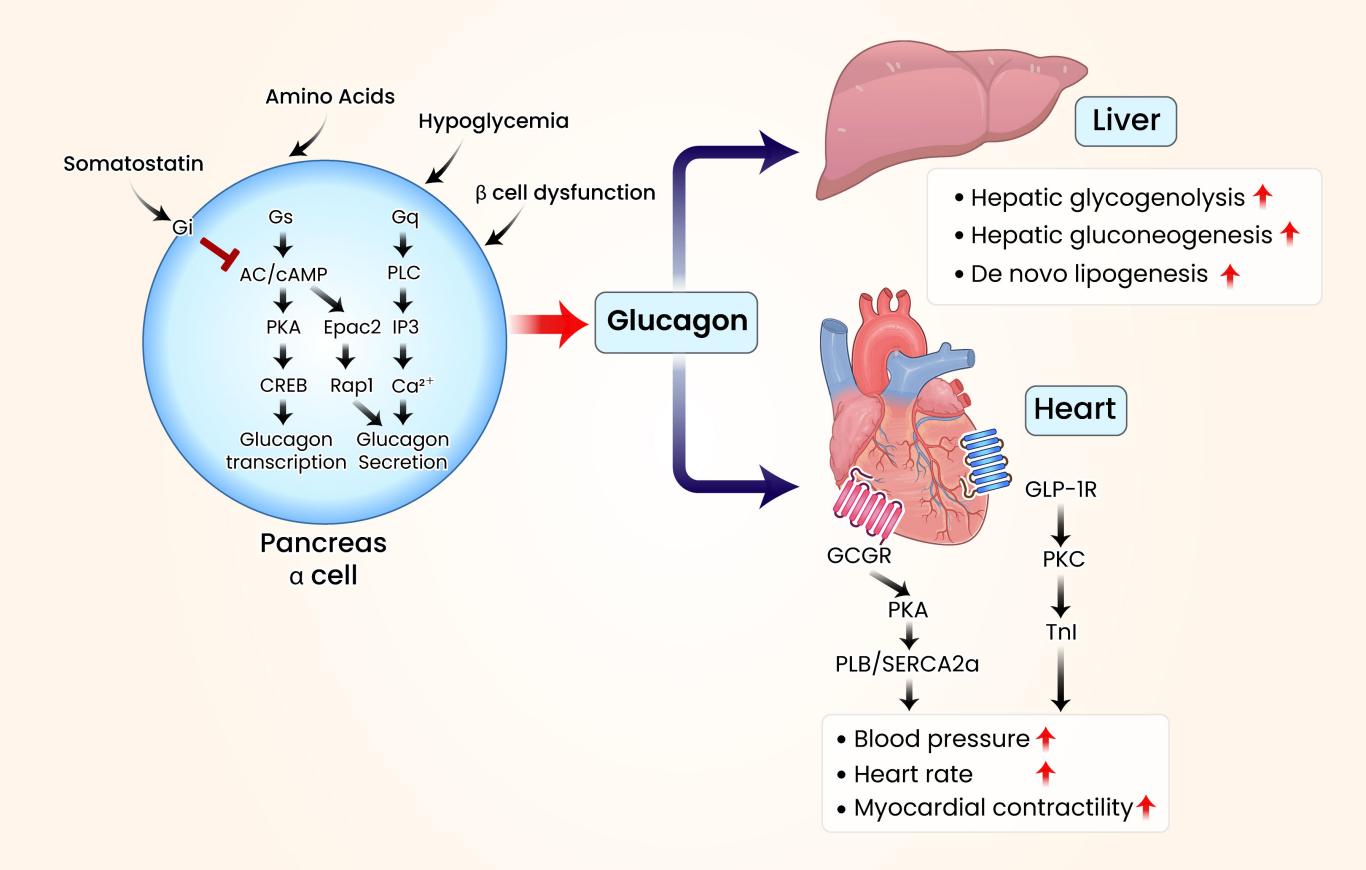
Schematic illustration of glucagon’s organ-specific metabolic effects in the liver and heart. In metabolic diseases such as diabetes, elevated blood glucose levels stimulate pancreatic β-cells to produce insulin, which lowers glucose levels. Insulin secretion is mediated by the activation of protein kinase A (PKA) through adenylate cyclase (AC) and cyclic adenosine monophosphate (cAMP). Conversely, pancreatic α-cells activate various G protein-coupled receptors (GPCRs) when blood glucose levels decline, triggering the AC/cAMP/PKA signaling cascade. This leads to increased glucagon synthesis through the regulation of cAMP response element-binding protein. Additional GPCR activation enhances glucagon secretion by increasing phospholipase C (PLC) and inositol triphosphate (IP3) activity or by upregulating exchange proteins activated by cAMP 2 (Epac2). Elevated glucagon levels raise blood glucose levels through various hepatic regulatory mechanisms. Most patients with diabetes exhibit elevated blood glucagon concentrations. When glucagon levels increase, glucagon receptors in the heart interact with glucagon signaling pathways, modulating the phosphorylation of cardiac Ca^2+^-regulatory and myofibrillar proteins. These interactions contribute to cardiac contractility, heart rate modulation, and conduction disturbances. Visual rendering by Biomedart based on author-designed content.

A comprehensive summary of these regulatory mechanisms, including upstream stimuli, receptor pathways, intracellular kinases, and their overall effects on α-cell glucagon secretion, is provided in **Table 1** and **Figure 1**.

**Table 1:** Key regulators of glucagon secretion.

Regulator	Source	Receptor /Pathway	Key Signaling Molecules	Glucagon Secretion
High Glucose	Postprandial state	β-/δ-cell paracrine signaling	↑ Insulin, ↑ Somatostatin → ↓ cAMP, ↓ Ca^2+^	Inhibition
Low Glucose	Hypoglycemia	Sympathetic activation	↑ cAMP, ↑ PKA, ↑ Epac2, ↑ Ca^2+^	Stimulation
Amino Acids	Protein intake/catabolism	GCGR feedback, amino acid sensors	mTORC1, AMPK (context-dependent)	Stimulation
Insulin	β-cell	Insulin receptor (IR)	PI3K-Akt signaling	Inhibition
Somatostatin	δ-cell	SSTR2 (on α-cell)	↓ cAMP, ↓ Ca^2+^	Inhibition
GABA	β-cell	GABAA receptor	Cl^-^ influx → Hyperpolarization	Inhibition
Zinc	Co-secreted with insulin	KATP channel modulation	↑ KATP activity	Inhibition
Epinephrine	Stress response	β-adrenergic receptor (β-AR)	↑ cAMP → ↑ PKA, Epac2	Stimulation
GLP-1	Gut (incretin)	GLP-1R (direct or indirect)	↓ cAMP, ↑ Insulin	Inhibition
GIP	Gut (incretin)	GIP receptor	↑ cAMP	Stimulation
Vasopressin	CNS/Hypothalamus	V1bR (Gq-coupled)	↑ PLC → ↑ IP3 → ↑ Ca^2+^	Stimulation

This table summarizes the major regulators of glucagon secretion in α-cells, including extracellular signals, receptors, and downstream signaling pathways.

↑, increase; ↓, decrease.

The symbol “→” denotes a biological or mechanistic pathway, indicating a directional relationship such as "leads to," "results in," or "promotes." It is used to show the downstream effects or progression within a process.

## 4 Disease regulation by glucagon

### 4.1 Type 1 and type 2 diabetes mellitus (T1DM and T2DM)

Glucagon receptor antagonists have effectively lowered blood glucose and HbA1c levels in patients with diabetes (26). As a crucial regulator of glucose homeostasis, glucagon stimulates hepatic gluconeogenesis and glycogenolysis, enabling rapid glucose mobilization during hypoglycemia (8, 22). In diabetes, disrupted cAMP signaling leads to impaired insulin secretion and excessive glucagon release (24). In T1DM, the lack of endogenous insulin eliminates its inhibitory effect on pancreatic α-cells, leading to increased glucagon secretion even during hyperglycemia (27). In T2DM, α-cells resist insulin-mediated paracrine suppression, resulting in elevated basal and postprandial glucagon levels (28). This plays a significant role in both fasting and postprandial hyperglycemia.

### 4.2 Obesity and energy balance

Beyond regulating glucose, glucagon also suppresses appetite through the liver–vagal nerve–hypothalamic axis and encourages thermogenesis by activating brown adipose tissue, thereby aiding weight loss (8, 29, 30). Glucagon has been shown to decrease food intake in rodent models (31, 32). Conversely, glucagon antibodies have been associated with increased food intake, highlighting their anorexigenic effect (33). Furthermore, earlier research suggested that increasing glucagon in obese mice reduces appetite and slows weight gain, indicating that glucagon may help regulate energy balance and obesity, not just raise blood sugar (34).

### 4.3 Lipid metabolism and energy expenditure

In lipid metabolism, glucagon promotes lipolysis, boosts fatty acid oxidation and ketone body production, and inhibits hepatic lipogenesis to prevent lipid accumulation. In addition to its metabolic effects, glucagon also increases oxygen consumption, as shown in early studies from the 1950s (16). Long-term glucagon treatment did not directly cause weight loss in animal models; however, weight reduction occurred due to increased energy expenditure (34). This effect is partly mediated by the upregulation of fibroblast growth factor 21 (FGF21), which activates the hepatic farnesoid X receptor (FXR), further increasing fatty acid oxidation and overall energy expenditure (35, 36).

### 4.4 Cardiovascular diseases

Although glucagon is mainly known for its metabolic effects, high doses used in pharmacological settings have been shown to temporarily raise blood pressure, heart rate, and myocardial contractility in preclinical studies (37–41). Based on these findings, glucagon has been used clinically as an emergency treatment for hypertension caused by β-blocker or calcium channel blocker overdose.

Glucagon exerts distinct cardiovascular effects by modulating cardiac contractility and conduction. It enhances myocardial performance through a positive inotropic and chronotropic response mediated by cAMP/protein kinase A (PKA) signaling. These effects lead to the phosphorylation of L-type calcium channels (LTCC), troponin I (TnI), and phospholamban (PLB), thereby enhancing calcium regulation and cardiac output (5, 42–45). However, cardiac response to glucagon can vary among species, heart regions, and disease states, depending on the distribution of glucagon receptors. GCGR agonists have shown promise in improving heart function in certain models of heart failure.

### 4.5 NAFLD and CKD

Hyperglucagonemia, characterized by elevated plasma glucagon levels, is a well-known feature of various metabolic disorders, including NAFLD and CKD (27, 46, 47). Although glucagon was historically regarded as a counter-regulatory hormone to insulin, it is now recognized as a key factor in the disruption of glucose and lipid metabolism in these conditions.

#### 4.5.1 Glucagon in NAFLD

Under physiological conditions, glucagon promotes hepatic glycogenolysis, gluconeogenesis, and fatty acid oxidation, while inhibiting *de novo* lipogenesis through receptor-mediated signaling (48). However, in the context of NAFLD and T2DM, hepatocytes exhibit impaired sensitivity to glucagon—a phenomenon referred to as hepatic glucagon resistance—which leads to compensatory hypersecretion of glucagon from pancreatic α-cells (49). In individuals with NAFLD, the liver may become resistant to glucagon, resulting in increased glucagon levels (hyperglucagonemia) as a compensatory response (8, 33). As a consequence, the lipid-lowering effects of glucagon are blunted, promoting hepatic triglyceride accumulation and persistent hyperglycemia (49). Additionally, glucagon-driven amino acid breakdown can lead to muscle wasting, providing substrates for gluconeogenesis and thereby further maintaining hyperglycemia (50).

#### 4.5.2 Glucagon in CKD

Glucagon contributes to renal physiology by regulating potassium homeostasis across nephron segments, a function that becomes increasingly relevant in the context of diabetic kidney disease (DKD) (51). In particular, altered potassium handling in DKD has been associated with progressive renal dysfunction and may exacerbate glucagon-mediated metabolic stress (52, 53). Additionally, the kidneys play a crucial role in glucagon clearance through glomerular filtration and tubular degradation, thereby maintaining systemic glucagon balance. In CKD, impaired renal function reduces glucagon elimination, resulting in elevated circulating glucagon levels that are independent of pancreatic output (54). Thus, this condition worsens hyperglycemia and insulin resistance, which may contribute to the development or progression of hepatic steatosis in CKD patients. Interestingly, in NAFLD, a paradox emerges: despite elevated plasma glucagon levels, hepatic lipid oxidation is blunted due to hepatic glucagon resistance (49).

## 5 Discussion

**Figure 2:**
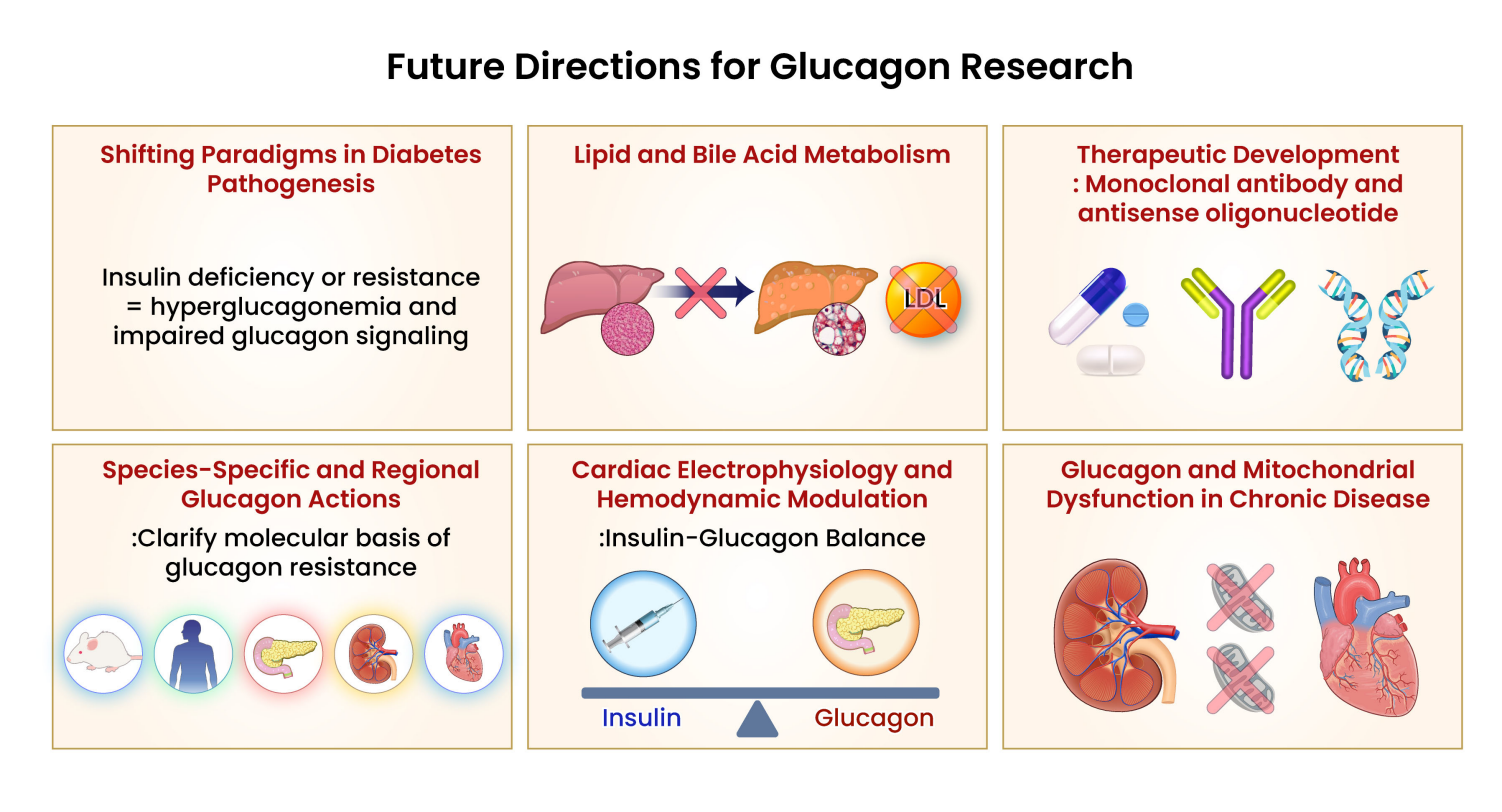
Conceptual summary of systemic glucagon dysregulation and its contribution to multi-organ metabolic dysfunction. Glucagon signaling is increasingly recognized as a central component in the pathophysiology of metabolic diseases, including type 2 diabetes mellitus (T2DM), non-alcoholic fatty liver disease (NAFLD), and chronic kidney disease (CKD). Under these conditions, mechanisms such as hepatic glucagon resistance, impaired renal clearance, and α-cell dysregulation contribute to persistent hyperglucagonemia. These alterations exacerbate hyperglycemia, promote hepatic lipid accumulation, and disrupt amino acid homeostasis. Emerging research highlights the need for targeted modulation of glucagon pathways, incorporating strategies that address tissue-specific resistance, balance α-cell function, and minimizing adverse systemic effects. Future therapeutic directions may involve dual receptor agonists, tissue-selective modulators, and humanized models that account for species-specific variability in glucagon receptor expression. Visual rendering by Biomedart based on author-designed content.

Glucagon is now recognized as a systemic hormone implicated in a broad range of metabolic and cardiovascular diseases. This review synthesizes emerging evidence on glucagon’s regulatory mechanisms, multi-organ effects, and translational implications, positioning glucagon as a central pathophysiological mediator beyond glycemic control (**Figure 2**).

### 5.1 A new perspective on the pathogenesis of diabetes: beyond insulin deficiency

While diabetes has traditionally been characterized by insulin deficiency or resistance, accumulating evidence suggests that hyperglucagonemia and impaired glucagon signaling are equally critical contributors to its pathogenesis. The glucagon-centric hypothesis, first proposed by Unger et al., posits that dysregulated glucagon secretion constitutes a common pathogenic mechanism in both type 1 and type 2 diabetes. Notably, studies using glucagon receptor–deficient (GCGR^-^/^-^) mouse models have demonstrated that inhibiting glucagon action can ameliorate hyperglycemia even in the absence of insulin (27, 55). We propose that chronic hyperglucagonemia and tissue-specific glucagon resistance represent not secondary phenomena, but primary upstream drivers of organ dysfunction in diabetes. This expands the pathophysiological paradigm from an insulin-centric to a glucagon-centric network failure model, with implications extending beyond glycemic control.

### 5.2 Emerging roles in lipid and bile acid metabolism

Beyond glycemic regulation, glucagon plays a multifaceted role in lipid and bile acid metabolism. Studies show that glucagon indirectly controls bile acid synthesis, absorption, and excretion by activating farnesoid X receptor (FXR) and increasing hepatic SHP1 expression (56). Additionally, glucagon seems to lower circulating LDL cholesterol by influencing PCSK9 or the LDL receptor through Epac2–Rap1 signaling (57). These findings highlight the promising therapeutic potential of glucagon-based strategies in treating dyslipidemia and metabolic liver disease, although further research is needed.

### 5.3 Challenges and innovations in therapeutic development

Numerous glucagon receptor (GCGR)–targeting compounds—including small molecules, monoclonal antibodies, and antisense oligonucleotides—have entered clinical development. However, their clinical utility remains limited due to heterogeneous metabolic effects and adverse outcomes, such as elevated alanine aminotransferase (ALT), increased low-density lipoprotein cholesterol (LDL-C), and weight gain (55). To address these limitations, several agents have progressed into early-phase clinical trials. For instance, volagidemab (REMD-477), a monoclonal antibody targeting GCGR, has been shown to reduce insulin requirements and lower mean daily glucose by approximately 27 mg/dL over 24 hours in individuals with type 1 diabetes, without increasing the risk of hypoglycemia (58, 59). Similarly, the small-molecule antagonist LY2409021 achieved HbA1c reductions of up to 0.83% over 12 weeks in patients with type 2 diabetes, although it was associated with mild, reversible elevations in liver enzymes (60, 61). To improve therapeutic efficacy while minimizing side effects, combinatorial strategies—particularly dual agonists that target both the glucagon and GLP-1 receptors—are under active investigation. In addition, machine learning–driven peptide design is emerging as a novel approach to optimize pharmacologic profiles and tissue specificity (62).

### 5.4 Species and region-specific glucagon actions: a translational challenge

One major obstacle in glucagon research is the species-specific differences in glucagon receptor expression and function, which make it difficult to apply findings from animal studies to humans. Future research should include humanized receptor models and examine the organ- and region-specific effects of glucagon to develop more accurate, tissue-specific treatments.

### 5.5 Cardiac electrophysiology and hemodynamic modulation

Glucagon’s role in cardiac health is complex. At high doses, glucagon has been associated with arrhythmias and conduction abnormalities; in contrast, physiologically controlled doses may help stabilize cardiac electrophysiology. This duality is fundamental in heart failure patients on insulin therapy, which has been associated with arrhythmogenic risk. Glucagon-based strategies may offer protective effects, particularly when an optimal insulin–glucagon balance is maintained by the patient’s hemodynamic profile.

### 5.6 Glucagon and mitochondrial dysfunction in chronic disease

Emerging data from db/db mouse models suggest that chronic hyperglucagonemia may impair hepatic mitochondrial function, thereby contributing to multi-organ metabolic deterioration (63). In contrast, human studies in individuals with hepatic steatosis have shown that baseline hepatic mitochondrial function is relatively preserved and can be significantly enhanced by glucagon stimulation. These findings suggest that glucagon functions as a dynamic modulator of mitochondrial activity, and this regulatory axis remains functional during the early stages of metabolic disease. However, disruption of this crosstalk in advanced disease may contribute to the progression toward metabolic failure (64). Further mechanistic studies are needed to clarify how glucagon-induced mitochondrial stress contributes to the progression of diabetes and its complications.

### 5.7 Future perspectives

While the systemic effects of glucagon are increasingly recognized, several important questions remain. First, the mechanisms of hepatic glucagon resistance and the resulting compensatory hyperglucagonemia require further study, particularly regarding their contribution to metabolic complications beyond glycemic control. Understanding how these alterations influence cardiovascular, renal, and hepatic dysfunction in diabetes remains a key research priority.

Second, the functional role of glucagon in mitochondrial metabolism—especially its potential to modulate oxidative capacity, bioenergetics, or stress responses—has not been fully elucidated. Clarifying these mechanisms may provide new insight into how glucagon contributes to disease progression or resolution in chronic metabolic states.

Third, species- and tissue-specific variability in glucagon receptor expression poses a major translational challenge. Future research must incorporate human-relevant models to better understand compartmentalized glucagon signaling and to enable therapeutic precision.

In parallel, we plan to evaluate whether targeting the glucagon axis may offer protective or disease-modifying effects in models of diabetes-related organ damage. These studies will assess both the risks and benefits of modulating glucagon action across different metabolic tissues.

Collectively, these investigations aim to refine our understanding of glucagon biology and to explore its therapeutic potential in mitigating multi-organ complications of diabetes.

## 6 Conclusion

Glucagon, historically regarded solely as a counter-regulatory hormone to insulin, is now recognized as a multifaceted regulator of metabolic homeostasis. In chronic metabolic conditions such as T2DM, NAFLD, and CKD, glucagon signaling is often dysregulated, contributing to disease progression through mechanisms that extend beyond hepatic glucose production. The evolving concept of glucagon resistance—particularly hepatic resistance coupled with compensatory α-cell hyperactivity—underscores the complexity of this hormonal axis in systemic metabolic control.

While therapeutic targeting of the glucagon pathway holds promise, clinical challenges such as dyslipidemia, hepatic stress, and amino acid imbalance must be carefully addressed. The dual nature of glucagon—as both a pathogenic driver and a therapeutic agent—demands precision-based strategies that account for tissue-specific responses and disease context.

Future glucagon-directed therapies will require a detailed understanding of systemic effects, receptor-specific signaling, and inter-organ feedback regulation. Continued investigation into α-cell biology, glucagon receptor pharmacology, and disease-specific modulation will be essential for developing effective and safe interventions. These insights emphasize the importance of reevaluating glucagon not only as a metabolic hormone, but also as a potential upstream driver of multi-organ complications in diabetes. Chronic alterations in glucagon secretion—commonly observed in patients with diabetes—may contribute to cardiovascular, renal, and hepatic dysfunction beyond glycemic control. This conceptual framework provides a foundation for future translational research into glucagon-based interventions targeting cardiometabolic disease.
